# Re-Envisioning Aging in the Right Place to Disrupt Homelessness for Older People

**DOI:** 10.1093/geroni/igab046.1127

**Published:** 2021-12-17

**Authors:** Rachel Weldrick, Sarah Canham, Joyce Weil

## Abstract

Recent developments in the aging-in-place literature have recognized the significance of aging-in-the-right-place. That is, aging in a place that supports an individual’s unique values, vulnerabilities, and lifestyles. This symposium will build upon existing research by critically examining the potential for older persons with experiences of homelessness (OPEH) and/or housing insecurity to age-in-the-right-place. Presenters will include interdisciplinary researchers with a diversity of perspectives stemming from gerontology, social work, and environmental design. The symposium will begin with Weldrick and Canham presenting a conceptual framework for aging-in-the-right-place that has been developed to outline indicators relevant to OPEH and housing-insecure older people. Elkes and Mahmood will then discuss findings from a study of service providers working with OPEH to consider the relative benefits and challenges of temporary housing programs. Following, Brais and colleagues will present findings from an environmental audit, developed as a novel assessment tool to evaluate the accessibility and physical design of housing programs for OPEH. A final presentation by Kaushik and Walsh will highlight findings from a photovoice study on perspectives of aging-in-the-right place among OPEH during the Covid-19 pandemic. Joyce Weil, an expert in measurement of person-place fit and life course inequalities, will discuss the implications of these papers and reflect on the potential for the aging-in-the-right-place framework to address the diverse needs of the growing population of OPEH through policy and practice. Together, the participants of the symposium will advance this emerging scholarship using a wide range of methods and perspectives.

